# Methods of Disinfecting Stethoscopes: Systematic Review

**DOI:** 10.3390/ijerph17061856

**Published:** 2020-03-13

**Authors:** Margherita Napolitani, Daiana Bezzini, Fulvio Moirano, Corrado Bedogni, Gabriele Messina

**Affiliations:** 1 Post Graduate School of Public Health, University of Siena, 53100 Siena; Italy; napolitani2@student.unisi.it; 2 Department of Life Sciences, University of Siena, 53100 Siena, Italy; daiana.bezzini@unisi.it; 3 General management, LHA Sardinia, 07100 Sassari, Italy; moirano1952@gmail.com; 4 Medical and General Management, S. Croce e Carle Hospital, 12100 Cuneo, Italy; bedogni.c@ospedale.cuneo.it; 5 Department of Molecular and Developmental Medicine, University of Siena, 53100 Siena, Italy

**Keywords:** stethoscope, healthcare-associated infections, copper, chlorhexidine, UV-LED, triclosan, isopropyl alcohol, benzalkonium, 90% ethanol, sodium hypochlorite

## Abstract

The aim of this systematic review was to investigate the effectiveness of various disinfection methods available for stethoscopes. In March 2019, we performed a search in PubMed and Scopus using the search terms: “reducing stethoscopes contamination” and “disinfection stethoscopes”; the Mesh terms used in PubMed were “Decontamination/methods” or “Disinfection/methods” and “Stethoscopes/microbiology”. Selection criteria were: English language; at least one disinfection method tested. A total of 253 publications were screened. After title, abstract, and full-text analysis, 17 papers were included in the systematic review. Ethanol at 90%, Ethanol-Based Hands Sanitizer (EBHS), triclosan, chlorhexidine, isopropyl alcohol, 66% ethyl alcohol, sodium hypochlorite, and benzalkonium chloride have been proven to lower the presence of bacteria on stethoscopes’ surfaces. In addition, alcohol wipes show effective results. A wearable device emitting ultraviolet C by Light-Emitting Diode (LED) resulted efficacious against common microorganisms involved in Healthcare Associated Infections. The cover impregnated with silver ions seemed to be associated with significantly higher colony counts. Instead, copper stethoscopes surface reduced bacterial load. The disinfection of stethoscopes appears to be essential. There are many valid methods available; the choice depends on various factors, such as the cost, availability, and practicality.

## 1. Introduction

Healthcare-associated infections (HAI) are a significant public health problem worldwide [1,2], with significant negative consequences, including impairment of patients’ health, mortality, and longer hospitalization, with the need for longer treatment and the associated higher costs [3,4]. In Europe, HAI causes 16 million extra days of hospitalization and 37,000 attributable deaths, resulting in associated costs of about 7 billion € per year [5]. It has been shown that more than 32% of HAI are avoidable [6,7] through hand washing and proper disinfection of medical devices between patients. The hands of healthcare professionals are the main vehicle for the transmission of microbes [3,8], but it is known that also contaminated medical devices (stethoscopes, otoscopes, and thermometers) can transmit microorganisms to patients [1,2,5]. In particular, stethoscopes, which are considered the symbol of medicine, are widely used medical devices but, if they are not disinfected, they can cause cross-contamination [9,10]. Unfortunately, although these instruments are in direct contact with many patients every day, their proper disinfection is not an established practice [2,11,12]. In addition, although the importance of careful disinfection of these tools has been repeatedly suggested [3,9,11,13], their disinfection is not yet common practice. For this reason, the US Centres for Disease Control and Prevention (CDC) published medical equipment disinfection guidelines to minimize the risk of cross infections. These guidelines follow the Spaulding classification, categorizing medical devices for the type of contact for which are used (critical, semicritical, non-critical items). Instruments coming into contact with sterile tissue or in the vascular system (surgical instruments, cardiac catheters, implants, etc.) must be steam sterilized or, if heat sensitive, sterilized with Ethylene Oxide, hydrogen peroxide plasma, liquid chemical sterilizers. Semi-critical items which come into contact with the mucous membranes or with the skin that is not intact (for example, endoscopes, laryngoscopes, esophageal probes items) require high-level disinfection with hydrogen peroxide, ortho-phthalaldehyde, and peracetic acid with hydrogen peroxide, etc. Non-critical objects which come into contact with intact skin but not with mucous membranes (bedpans, blood pressure cuffs, crutches, etc.) require low-level disinfection [14].

In the majority of cases, stethoscopes are used on intact skin (noncritical contact) and so the recommendations suggest to disinfect it for “each patient or once daily or once weekly”, whereas in the case of semi-critical contact, as in the case of use on skin that is not intact (e.g., trauma), stethoscopes should be disinfected “before use on each patient” [14,15]. A recent study that anonymously evaluated the methods and frequency of cleaning of stethoscopes by the healthcare providers demonstrated that stethoscopes were disinfected following CDC guidelines in less than 4% of respondents and were not disinfected at all in 82% of respondents [15]. Additionally, a 2015 systematic review, which investigated the possible risks of infections transmitted to patients by uncleaned stethoscopes, found a mean level of contamination in excess of the French Normalization standard for cleanliness (which equates to <5 colony-forming units per cm^2^ or <20 CFU per membrane) in all studies in which contamination levels were quantified [7]. All this despite, over the years, numerous studies have demonstrated the presence of microbes on the surfaces of stethoscopes, which have principally been commensal skin micro-organisms, but also potential pathogens, such as *Methicillin-Sensitive Staphylococcus aureus, Methicillin-Resistant Staphylococcus aureus (MRSA), Enterococcus, Klebsiella, Acinetobacter, vancomycin-resistant enterococci,* and *Clostridium difficile* [7,13].

There are many disinfection methods for stethoscopes. Some refer to the use of “classic” disinfectants, others to the use of new methods. A revision updating the appearance of the stethoscope disinfection is missing. The aims of this systematic review were: (I) to specify the different types of disinfectants available; and (II) to compare their disinfective effectiveness. 

## 2. Materials and Methods 

In March 2019, we performed a systematic review in order to evaluate and compare various disinfection methods. We looked for original peer-reviewed papers in the electronic database PubMed (MEDLINE) and Scopus. The key search terms were “reducing stethoscopes contamination” and “disinfection stethoscopes”. The Mesh terms used in PubMed were “Decontamination/methods” OR “Disinfection/methods” AND “Stethoscopes/microbiology”. The database searches were performed by two independent authors (M.N. and G.M.). For the assessment of methodological quality, we used standardized Critical Appraisal Tools from the Joanna Briggs Institute (JBI): Checklist for Analytical Cross Sectional Studies, for Cohort Studies and for Randomized Controlled Trials [16]. Risk of bias was assessed independently by two authors (M.N. and G.M.), using the previously described risk of bias tools. For cross-sectional studies, the tool used asked the following questions: “(1) Were the criteria for inclusion in the sample clearly defined? (2) Were the study subjects and the setting described in detail? (3) Was the exposure measured in a valid and reliable way? (4) Were objective, standard criteria used for measurement of the condition? (5) Were confounding factors identified? (6) Were strategies to deal with confounding factors stated? (7) Were the outcomes measured in a valid and reliable way? 8) Was appropriate statistical analysis used?”. As far as the cohort studies were concerned, the questions to be answered were: “(1) Were the two groups similar and recruited from the same population? (2) Were the exposures measured similarly to assign people to both exposed and unexposed groups? (3) Was the exposure measured in a valid and reliable way? (4) Were confounding factors identified? (5) Were strategies to deal with confounding factors stated? (6) Were the groups/participants free of the outcome at the start of the study (or at the moment of exposure)? (7) Were the outcomes measured in a valid and reliable way?; (8) Was the follow up time reported and sufficient to be long enough for outcomes to occur? (9) Was follow up complete, and if not, were the reasons to loss to follow up described and explored? (10) Were strategies to address incomplete follow up utilized?; (11) Was appropriate statistical analysis used?”

Finally, for the randomized controlled trials, the questions were: “(1) Was true randomization used for assignment of participants to treatment groups? (2) Was allocation to treatment groups concealed? (3) Were treatment groups similar at the baseline? (4) Were participants blind to treatment assignment? (5) Were those delivering treatment blind to treatment assignment? (6) Were outcomes assessors blind to treatment assignment? (7) Were treatment groups treated identically other than the intervention of interest? (8) Was follow up complete and if not, were differences between groups in terms of their follow up adequately described and analyzed? (9) Were participants analyzed in the groups to which they were randomized? (10) Were outcomes measured in the same way for treatment groups? (11) Were outcomes measured in a reliable way? (12) Was appropriate statistical analysis used?” The possible answers were: Yes, no, unclear, and not applicable. For each study, an overall assessment was required: Studies to be included, excluded, or need to seek further info [16]. The two reviewers, after analysis, assessed that all studies should be included and considered that all studies were low or medium risk of bias. Any disagreements that arose were resolved by discussion. There was no need for recourse to a third Reviewer. The inclusion criteria were: (1) Studies that measured the effectiveness of at least one method of disinfecting the stethoscopes; (2) disinfection methods not tested on stethoscopes (also associated with other devices); (3) prospective or cross-sectional study; (4) English language; (5) studies published between 1997 and 2018. The exclusion criteria were: (1) Surveys of stethoscope disinfection habits and/or bacteriological evaluation of stethoscopes in the absence of quantitative evaluation of the effectiveness of a disinfection method; (2) not disinfection methods tested or disinfection methods not tested on stethoscopes; (3) studies with results not clear, sampling not clear; (4) studies testing for contamination of disposable covers or single use stethoscopes. All studies consistent with the selection criteria were included in the research. For each study included, we extracted the country, the year, the setting, the study design, the aim, the number of stethoscopes analyzed (sample), the characteristics of the sample, the reduction percentage of contamination, the microbial contamination before and after disinfection and the findings. The results were reported by the studies in the form of the mean number of Colony-Forming Unit (CFU), the median (IQR) number of CFU, the CFU total count and the presence or absence of at least one colony.

## 3. Results

**Figure 1:**
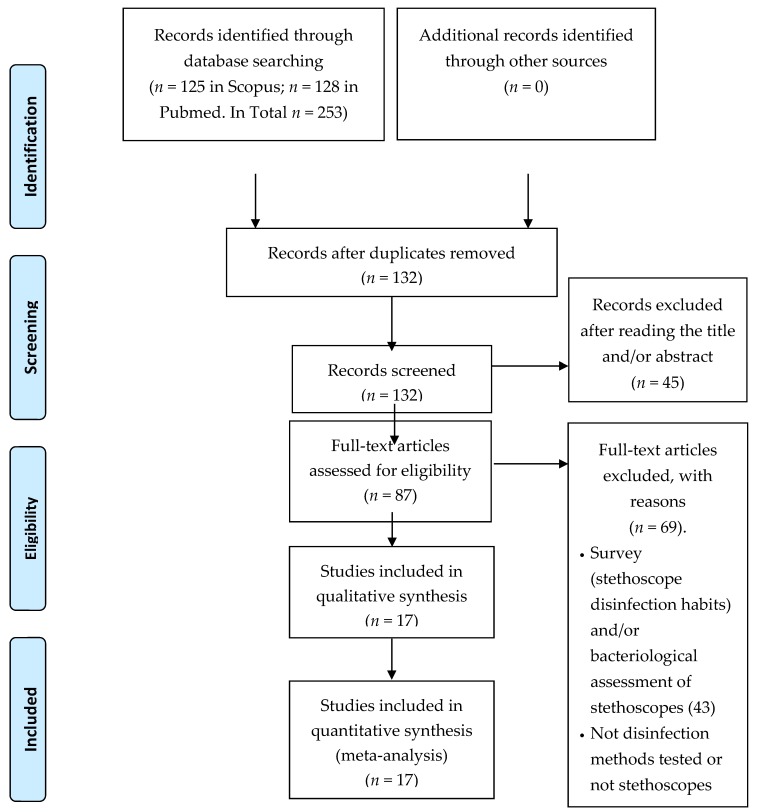
Preferred Reporting Items for Systematic reviews and Meta-Analyses (PRISMA) 2009 Flow diagram illustrating the process of selection of the eligible articles used in this paper.

The literature search yielded 133 papers. We have analyzed the title of these publications, excluding 16 studies because they were written in Portuguese, Polish, Dutch Turkish, Spanish, or in French, and 29 others because they were not in line with the aim of the study. Moreover, 70 studies were commentaries or full articles not in line with the aim of the study and we have excluded them after a careful examination of the abstracts and of the full texts. Finally, we detected 17 publications [3,5,6,9,11,13,17,18,19,20,21,22,23,24,25,26,27]. Among these, we included four letters to the editor (Figure 1).

These studies, published between 1997 and 2018 and carried out in France, India, Italy, Nepal, Mexico, the United Kingdom, and the United States, tested various disinfection methods. The main results of our review are shown in Table 1.

In 1997, 40 randomly selected stethoscopes were analysed on the diaphragm and under the edge before and after disinfection with various chemical antiseptics. A mean of 158 ± 33 and of 289 ± 54 Colony-Forming Units (CFUs) were found respectively on stethoscope diaphragm and on the rims before disinfection. Sodium Hypochlorite (NaOCl), 70% Isopropyl alcohol (IPA), and Benzalkonium Chloride (BAK) significantly reduced bacterial load, but IPA was the most effective in the rim area. It reduced the CFU to 0.2 ± 0.2 (*p* = 0.02) on the stethoscope diaphragm and to 2.2 ± 1.5 (*p* = 0.01) on the area of the rim. In contrast, soap and water reduced bacterial load, but not in a statistically significant way (47 ± 28 CFU *p* = 0.11 on the diaphragm and 95 ± 48 CFU *p* = 0.09 on the rims) [11]. 

Parmar et al. [26] found that 66% ethyl alcohol was an effective disinfectant. For each of the 100 stethoscopes used in their study, four samples were taken: One before cleaning (Group A), one immediately after cleaning (Group B), one after five days without cleaning (Group C), and one five days after cleaning once a day (Group D). In the Group A, 90% of the stethoscopes were contaminated. Group B and Group D both led to a significant reduction in the contamination rate of stethoscopes to 28% and 25%, respectively. In Group C, the rate of contamination was 95%. No statistically significant differences were found between Group A and C.

Datta et al. [27] proved that IPA is significantly effective in disinfection. A total of 100 stethoscope diaphragms were analyzed before and after cleaning with IPA. Fifty-six membranes were found colonized before disinfection, while after disinfection, there were five colonized membranes (*p* < 0.001).

Raghubanshi et al. [13] proved that both 90% ethanol and IPA are equally effective in decontaminating the diaphragm of the stethoscope. The baseline median (IQR) was 22 (7–48) CFU for IPA and 15.5 (7–31) for 90% Ethanol, the post-cleaning median (IQR) was 0 (0–0) for both. Similar results were found by Alvarez et al. 2016. The baseline median (IQR) was 10 (3–43) CFU and zero (0–0) immediately after disinfection with IPA. With regards to recontamination after disinfection, significant differences between bacterial counts after disinfection with chlorhexidine or alcohol have not been found after 1 min without use. Among the chemical agents examined, only chlorhexidine prevented the recontamination of stethoscopes for at least four hours after disinfection [9]. 

Also, the use of Ethanol-Based Hand Sanitizer (EBHS) (gel or foam) was effective in disinfection [18,19,20]. In a study conducted by Grandiere-Perez et al. [18], before disinfection, 38 out of 40 stethoscopes examined were culture positive with a mean of 29.9 CFU per culture plate. After disinfection, the mean was 1.1 CFU *p* < 0.001. Schroeder et al. [20] found a mean of 28.4 CFU (95% CI, 20.2–36.6), in the pre-wash sample, and a mean of 3.2 (95% CI, 1.8–4.6; *p* < 0.001) in the post-wash sample. Mehta et al. [19] found that both IPA wipes and EBHS was effective, but the reduction of the bacterial count with the wipes was significantly greater than with the EBHS (*p* = 0.001). Hill et al. [21] organized a poster campaign to raise awareness of the importance of cleaning stethoscopes and provided for the increased availability of alcoholic wipes. In addition, training interventions were organized at departmental meetings. By obtaining samples from the stethoscopes of elderly care doctors, they found a mean of 70 CFU at baseline and a mean of 59 and 41, respectively, at one and three months after the start of the campaign. Staphylococcus aureus colonies fell from 0.5 per stethoscope to 0.25 at one month but went up to 0.4 per stethoscope at three months. The mean of MRSA CFU decreased by 0.42 per stethoscope to 0.08 per stethoscope at one month. No MRSA colonies were found at three months. 

Moreover, wipes impregnated with benzalkonium can be used to clean stethoscopes. Leprat et al. [22] evaluated 105 stethoscopes. The number of CFU on one half of the diaphragm of the contaminated stethoscopes was between 1 and 10 in 34, 11 and 100 in 17 and >100 in 16. After cleaning, no stethoscope was contaminated (100% reduction).

With regards to physical methods of disinfection, Schmidt et al. [17] assessed the efficacy of antimicrobial copper stethoscope surfaces to reduce the bacterial concentration on the diaphragm, binaural tube and ear tubes. They collected stethoscopes from 21 clinical providers (14 health care providers of Paediatric Emergency Division (PedsED) and seven of an adult medical intensive care unit). Out of 32 stethoscopes, 276 samples were taken. The mean CFU on copper surfaces (three surfaces sampled) from stethoscopes used in the paediatric ED was 11.7 CFU/cm^2^. This concentration was significantly lower than the concentrations recovered from similar surfaces by control stethoscopes (127.1 CFU/cm^2^, *p* < 0.001). Considering separately the areas that were sampled, a mean value of 4 CFU/cm^2^ was found on the copper diaphragm, whereas a mean value of 16 CFU/cm^2^ was found on control diaphragm, but the difference between the two groups was not significant (*p* = 0.089). However, in the stethoscopes analyzed from the adult settings, this difference was found significant (mean of 5 CFU/cm^2^ on copper diaphragm and mean of 10 CFU/cm^2^ on control diaphragm *p* = 0.005). Another surface analyzed was the binaural tube for transmission of the sound from the bell to the ears. The difference between the two materials was found to be significant (*p* <  0.001). A mean of 2 CFU/cm^2^ was recovered from the copper binaural tube and a mean of 108 CFU/cm^2^ was found on control binaural tube. On the contrary, it was found a lower mean microbial burden (4 CFU/cm^2^) on the aluminium earplugs compared to that of the copper antimicrobial tubes (5 CFU/cm^2^); this difference achieved significance (*p* = 0.002). No significant difference was observed between the mean of CFU found on urethane rim of the control stethoscopes and of copper stethoscopes, neither in the paediatric setting (control: 302 CFU/cm^2^; *n* = 27 and copper stethoscopes: 317 CFU/cm^2^; *n* = 28) nor in the adult ward (control: 125 CFU/cm^2^; *n* = 14 and copper, 83 CFU/cm^2^; *n* = 14).

Another physical method of disinfection found was the use of UV. In a laboratory experiment, Messina et al. [6] found that also the use of a device emitting Ultraviolet C (UVC) light through a Light-Emitting Diode (LED) could reduce bacterial load on stethoscopes membrane surface. On 10 stethoscopes analyzed, the mean (±SD) number of CFU was 9.5 (±18.8) after treatment and 75.9 (±125.7) without treatment. The median number of CFU was 2.5 (IQR: 0–10.5) after treatment and 38 (IQR: 12.5–68.75) without treatment. The average CFU reduction between the two groups was 85.7% (*p* = 0.002). In another study, three repeated auscultations were carried out on a volunteer (with a total of ten contact points each). The first auscultation was used as a control, while the other two tested the disinfection capability of LEDs. The membrane was disinfected with alcohol, before and after each use of the stethoscope. A total of 104 CFU were found at baseline, while 12 CFU and 15 CFU were found after two independent tests (*p* < 0.001) [23].

In the laboratory, this device was effective in reducing the bacterial burden of *E. coli, S. aureus, P. aeruginosa* and *E. faecalis*. The reduction percentage was all above 85%. The median (and IQR) of *S. aureus* CFU on the control stethoscopes was 56 (51–64), and on those treated, it was 7 (5–8), *p* < 0.01; the median (and IQR) of E. Coli CFU was 35 (27–43) on the controls stethoscopes and 2 (1–3) on those treated (*p* < 0.01). With regards to *P. aeruginosa,* the median (IQR) was 39 (38–41) on the control, and 2 (2–3) *p* < 0.01 on the treated. Finally, for *E. faecalis*; Controls: 228 (198–261) and Treated: 33 (26–36), *p* < 0.01 [3]. 

Later, this device was also tested in a real context. In November 2016–May 2017, a cross-sectional study was conducted in a private clinic to test the efficacy of a device emitting UVC light for disinfecting stethoscope membranes. The number of tests of stethoscopes treated with the device was 116 out of 272. Untreated sample had a mean contamination of 132.2 CFU (95% CI, 106.08–157.57), while treated sample had a mean contamination of 6.9 CFU (95% CI, 2.7–13.46) (*p* < 0.001). These results suggest that, in a real environment, the UV-C device can efficiently and effectively disinfect stethoscope membranes, even if they are highly contaminated. Overall, the percentage reduction in CFU was 94.8 (95% CI, 91.3–97.7) [26].

To disinfect stethoscopes and also various instruments used in hospital settings, such as telephone handsets and computer keyboards, a putty compound with a malleable-elastic consistency that allows it to adhere and to remove the dirt, was also found to be effective in combination with disinfecting activity. This compound was composed primary by ethanol, water, guar, dyes, and odorants. In a cross-over study involving an Italian teaching hospital, the microbial contamination of stethoscopes membranes was evaluated before and after cleaning with this compound. A total of 35 stethoscopes were analyzed: The mean (±SD) of CFU at 36 °C was 168.4 ± 304.7 before cleaning and no growth was measured after cleaning. The mean ± SDof CFU at 22 °C was 167.2 ± 367, with a reduction of 100% after cleaning (0 CFU found) [5].

Wood et al. [24] evaluated the utility of diaphragms’ cover infused with silver ions. They analyzed 74 stethoscopes (37 with cover and 37 without cover). At the time of culture, each health care worker completed a short survey, in which they reported, in addition to their role, whether they had used the cover or not, for how long, the type of disinfectant used to clean the stethoscope, and with what frequency. The CFUs were significantly lower in uncovered stethoscopes (mean 71.4 CFU) than those short-term cover use (<1 week as recommended by the manufacturer): Mean 264.5 CFU. When the cover was used for more than a week, the mean of CFU found was 335.6.

## 4. Discussion

All the chemical disinfectants proved to limit the bacterial presence on stethoscope surfaces [9,11,13,18,19,20,21,22]. There are various formulations (liquid, gel, foam, or putty) that can be used according to preferences and availability. The oldest study we found dates back to 1997, in which Marinella et al. [11] tested the effectiveness of isopropyl alcohol, sodium hypochlorite, benzalkonium chloride, and soap and water (Acute-Kare Handwash, Calgon Vestal Labs, St Louis, Mo). Washing with soap and water was the only one that did not prove significantly effective in reducing the bacterial load on stethoscope diaphragm and under the rim. Among other three, isopropyl alcohol was most effective in disinfecting the rim area, probably because of the biochemical properties that allow it to spread more easily under the rim.

Other authors have evaluated the effectiveness of other chemical disinfectants. The EBHS was effective to significantly reduce the number of bacterial colonies on stethoscopes [18]. Clinicians could rub their hands with EBHS and then they could rub the stethoscope diaphragm between the hands in order to disinfect it stethoscopes [19,20]. This method was effective and reduced bacterial colonies including MRSA [20].

In this way, EBHS and IPA pads are very effective in reducing the bacterial load on stethoscopes surfaces, but, in developing countries, they are not easily available because of their cost. On the contrary, ethanol at 90% is extremely widespread and cheaper. In a study conducted in Nepal, Raghubanshi et al. [13] demonstrated that ethanol at 90% was effective like IPA pads in reducing bacterial load on stethoscope. These results allow developing countries to choose the most affordable alternative based on cost, with the security of using an equally effective product.

The use of disinfectant wipes is a practical and fast method for disinfecting stethoscopes and other authors have also evaluated their effectiveness [19,21,22]. Leprat et al. [22] demonstrated the effectiveness of wipes impregnated with benzalkonium against coagulase-negative staphylococci (CNS), CNS resistant to methicillin (MR-CNS) and methicillin-susceptible Staphylococcus aureus (MSSA). After cleaning, all contaminated stethoscopes showed no bacterial growth.

As part of a campaign to raise awareness of the importance of cleaning their stethoscopes, Hill et al. [21] displayed posters in places visible to doctors but not public and have increased the availability of alcoholic wipes. All these initiatives led to the disappearance of the MRSA colonies on stethoscopes surfaces, which is the problems caused by this pathogen causes. Mehta et al. [19] assessed and compared the effectiveness of EBHS and IPA pads: Both methods reduced bacterial contamination, but a single cleaning with an alcohol wipe was more effective than EBHS. The authors assumed that this was due to the mechanical effect of the pads, but concluded that, since the availability of wipes is lower than the spread of EBHS, the stable use of wipes may not be practical and feasible. In addition, the use of a single product for simultaneous cleaning of hands and stethoscope and its ubiquitous presence in the hospital could facilitate its inclusion in routine practice between patient examinations. With regard to chemical disinfectants, one aspect highlighted is recontamination of stethoscopes membranes after disinfection. While there were no significant differences between bacterial load immediately after disinfection with chlorhexidine or IPA, the use of chlorhexidine prevented the recontamination of stethoscopes for at least 4 h after disinfection, in fact the bacterial load found was significantly lower than that of the surfaces of stethoscopes disinfected with IPA. This could be due to the rapid evaporation of the alcohol, the effect of which is prolonged only if the surfaces remain immersed [9]. 

With regards to physical disinfection methods, a wearable device emitting UVC-LED was effective against common microorganism involved in healthcare-associated infections; In fact, this device was effective in reducing bacterial load of Escherichia coli, Staphylococcus aureus, Pseudomonas aeruginosa and Enterococcus faecalis. It was practical to use, and it could prevent cross contamination [3,6]. It has also been shown that the UVC-LED was still effective to reduce microbial contamination after prolonged use (more than 240 working hours). Since it can be attached to the pocket of the medical coat, the positive aspect of this device is that it can make the disinfection of the stethoscope an automatism repeated after each use [23]. 

Another good strategy involves the use of antimicrobial copper surfaces. It is well known that solid copper and alloys containing > 60% copper by weight have the property to kill bacteria to the touch [17,28]. This intrinsic antimicrobial activity of copper was confirmed for various surfaces [17,28]. By decreasing bacterial proliferation, the use of copper stethoscopes surfaces could prevent HAI [17].

As part of a broader study to assess environmental contamination in hospitals, the disinfectant efficacy of a putty compound based on ethanol, water and glycerine was confirmed. This putty could be used to disinfect not only stethoscopes but also telephone handsets, computer keyboards, and other objects [5]. 

Another physical method to prevent cross-infections could be the use of silver- infused cover. Antimicrobial stethoscope covers impregnated with silver ions have been designed to avoid surface contamination. Astonishingly, Wood et al. found that the bacterial load was greater in the surfaces of stethoscopes with cover than in those without cover, especially if the cover was used more than the time suggested by the manufacturer (one week). In fact, a prolonged use of covers appeared to result in even higher colony counts, regardless the cleaning agents used to disinfect. These unexpected results are perhaps due to the presence of the embossing that may protect bacteria from cleaning agents. However, the authors suggest the need to conduct further and larger studies to confirm these findings [23]. The cost/benefit ratio of the various disinfection methods is also worth mentioning. In addition to the cost of the chemical disinfectant or physical disinfection tool or to limit contamination, the cost of consumables (wipes, gauze, cotton, etc.) must also be considered. The cost of disposing of this special waste should not be disregarded. Even if they do not fall within the scope of our research, these are all considerations to be made when choosing the type of disinfection to be used

This research has several limits. There are no studies in the literature reporting a healthcare-associated infection caused by contact with contaminated stethoscope membrane. However, many studies have found that bacteria, including resistant microorganisms, are present on stethoscopes membranes and that methicillin-resistant Staphylococcus aureus can potentially survive up to nine days on stethoscopes [29]. In addition, two-way transfer of microorganisms, including pathogens, has been shown to be possible between the skin and the stethoscope [25]. For example, the same serotype O12 strain of Pseudomonas aeruginosa was found on the skin and stethoscope membrane [25]. Moreover, stethoscopes are contaminated as much as the dominant part of the hands and therefore it is possible that they can cause cross-contamination and HAI [30,31]. 

## 5. Conclusions

Proper disinfection of stethoscopes after each use can help to reduce cross-contamination and consequentially reduce the likelihood of HAI. There are many effective methods for disinfecting stethoscopes: Chemical methods include disinfection with IPA 90% ethanol, chlorhexidine, in liquid formulations, gels, or foams, and in the form of alcohol-soaked wipes. Physical methods include the use of UVC-LED devices and stethoscopes with antibacterial copper surfaces. Instead, the use of silver-infused cover does not appear to be effective, but further larger randomized trial may be needed to validate these results [24]. In conclusion, it is essential that health professionals understand the potential risks associated with poorly cleaned stethoscopes and therefore the importance of disinfecting them after each use. The choice of the method to use depends on effectiveness, availability, price, easy to use and common practice. The failure of disinfection methods does not seem to be due to the lack of effectiveness of what is used, but rather to a lack of regularity in the use of the products. An approach to disinfection with mechanisms that provide for automation and do not alter the activity of health care workers could be useful to maintain good sanitation performance over time.

## Figures and Tables

**Table 1:** Main characteristics of the studies included in the systematic review.

Author, Year	Country	Year	Setting	Study Design	Aim	Sample (*N*)	Sample Characteristics	Reduction (%)	Results	Findings
Messina et al. (2018)	Italy	Nov 2016–May 2017	Four wards of a private clinic	Cross-sectional study	To test the efficacy of a device emitting UVC light for disinfecting stethoscope membranes	272	*Stethoscopes*	94.8% (95% CI 91.3–97.7)	The mean number of CFU: Not Treated: 132.2 Treated: 6.9 (*p* < 0.001)	The UV-C device can efficiently and effectively disinfect stethoscope membranes, even if they are highly contaminated
Datta et al. (2018)	India	2015	Tertiary care hospital	Cross–sectional study	To determine the disinfectant efficacy of 70% IPA	100	*Stethoscopes*	96.2%	The mean number of CFU: Before disinfection: 65 After disinfection: 2, 5 Colonization: Before disinfection: 56 After disinfection: 5 (*p* < 0.001)	IPA is significantly effective in disinfection
Schimidt. et al. (2017)	U.S.A.		Tertiary care facilities (PedsED and AICU)	Structured prospective trial;	To assess the efficacy of antimicrobial copper stethoscope surfaces to reduce the bacterial concentration	32	*Stethoscopes*	90.8%	The mean number of CFU (PedsED) Three surfaces sampled: 11.7/cm^2^ Copper arm 127.1/cm^2^ Control arm (*p* < 0.001) Diaphragm: 4/cm^2^ Copper arm 16/cm^2^ Control arm (*p* = 0.09) The mean number of CFU (AICU) diaphragm: 5/cm^2^ Copper arm 10/cm^2^ Control arm (*p* < 0.01)	Copper surfaces proved to limit the concentration of bacteria on stethoscopes surface (not always statistically significant)
								75%		
								50%		
Messina G. et al. (2017) *Letter to Editor*	Italy	2015	Laboratories of University	Pilot study pre/post design	To test the efficacy of a device emitting UVC light through a LED to reduce bacterial load on stethoscopes surface	10	*Stethoscopes*	85.7%	The mean (±SD) number of CFU: Not Treated: 75.9 ± 125.7 Treated: 9.5 ± 18.8. The median (IQR) number of CFU: Not Treated: 38 (12,.5–68, 75) Treated: 2.5 (0–10.5). (*p* < 0.01)	-The device was effective and practical to use -It may be an advantage because of lack of resistance to UVC from micro-organism involved in HAI
Raghubanshi et al. (2017)	Nepal	Dec 2016–March 2017	Tertiary care hospital	Randomized blinded experimental study	To determine the effectiveness of 90% ethanol compared with isopropyl alcohol pads to reduce the bacterial load	108	*Stethoscopes*	100	The median (IQR) number of CFU: Before disinfection: -IPA 22.5 (7–48) -Ethanol 17.5 (7–31) After disinfection: -IPA 0 (0–0) (*p* < 0.001) -Ethanol 0 (0–0) (*p* < 0.001)	Both 90% ethanol and IPA are equally effective in decontaminating the diaphragm of the stethoscope
Alvarez MD et al. (2016)	Mexico	2013	Secondary care hospital/ Tertiary care hospital	Experimental, controlled blinded trial	To determine differences in recontamination of stethoscope membranes after being cleaned with chlorhexidine, triclosan or alcohol	370	*Stethoscopes*	100 (IPA)	Median (IQR) of CFU for study arms: baseline: 10 (3–42) IPA, at the time 0: 0(0–0) IPA, the residual effect at 4h: 8 (1–28) Triclosan, the residual effect at 4 h: 4 (0–17) Chlorhexidine, residual effect at 4h: 0 (0–1) Kruskal–Wallis Test: 133.2 (*p* < 0.001)	-Chlorhexidine prevented the recontamination of stethoscopes for at least 4 h after disinfection -No significant differences between the bacterial load of the chlorhexidine arm and one of the immediate effects of the isopropyl alcohol
Messina G. et al. (2016)	Italy	August 2015–March 2016	Laboratories of University	Cross-sectional study pre/post design	To test if the UVC LEDs are still effective to reduce microbial contamination after a prolonged use	1	*Stethoscopes*	85.6	The number of CFU: Not Treated: 104 CFU Treated (LED 16):15 CFU (*p* < 0.001) Treated (LED 18):12 CFU (*p* < 0.001)	UVC LEDs were still effective in disinfection after a prolonged use
Messina G et al. (2015)	Italy	n.r.	Laboratories of University	Cohort study	To test the efficacy of a device emitting UVC light for reducing bacterial load of E. Coli, S. Aureus, P. Aeruginosa and E. Faecalis	28	*Stethoscopes*	>85	Median (and IQR) of CFU: S. aureus: Not Treated: 56 (51–64) Treated: 7 (5–8) *p* < 0.01 E. coli: Not Treated: 35 (27–43) Treated: 2 (1–3) *p* < 0.01 P. aeruginosa: Not Treated: 39 (38–41) Treated: 2 (2–3) < 0.01 E. faecalis: Not Treated: 228 (198–261) Treated: 33 (25–36) *p* < 0.01	For all four species, statistically significant differences were found in CFU count after one UVC treatment
Grandiere-Perez et al. (2015) *Letter to Editor*	France	n.r.	Le Mans Hospital	Cross-sectional study pre/post design	To test the effectiveness of an EBHS to reduce the number of bacterial colonies on stethoscope diaphragms.	40	*Stethoscopes*	96.3	The mean number of CFU: Before disinfection: 29.9 per plate After disinfection: 1.1 per plate Colonization: Before disinfection: 38 out of 40 (95%); After disinfection: 22 out of 40 (55%); *p* < 0.001	The EBHS was effective to significantly reduce the bacterial load on stethoscopes
Messina G. et al. (2013)	Italy	n.r.	Hospital of Siena	Cross-sectional study pre/post design	-To evaluate the environmental contamination in Hospital Setting and to evaluate the efficacy of a putty compound (the main components: ethanol (29%), water (51%, guar (6%), glycerine (%))	35 37 27	*Stethoscopes* *Telephone handsets* *Computer keyboards*	>99	CFU total count: TBC at 36°: Before disinfection: 3368 After disinfection: 1 TBC at 22°: Before disinfection: 3678 After disinfection: 0	-Proper disinfection of medical devices is very important -The disinfecting technique used was effective in reducing bacterial load
Mehta et al. (2010)	U.S.A.	n.r.	Grady Memorial Hospital and Emory University Hospital Midtown	Cross-sectional study pre/post design	To test the efficacy of alcohol-based hand rubs to reduce bacterial load on stethoscope surfaces and to compare it with that of the isopropyl alcohol wipes	84	*Stethoscopes*	90	The median (IQR) number of CFU: Before disinfection: 34.5 (0–247) After disinfection: -Alcohol hand rub: 4 CFU (0–60) *p* < 0.001 no growth in 12 (20%) out of 60 - Alcohol wipe: 0 CFU (0–59) no growth in 17 (71%) out of 24 *p* = 0.001	-Both methods significantly reduced bacterial contamination - the alcohol wipes were more effective but less available
Schroeder et al. (2009)	U.S.A.	n.r.	A community-based hospital and 1 satellite family health center	Prospective, single-blinded study Pre/post Design	To test if clinicians can simultaneously disinfect stethoscope diaphragm and their hands with alcohol-based foam	92	*Stethoscopes*	88.7	The mean number of CFU: Before disinfection:28.4 (95% CI, 20.2–36.6) After disinfection: 3.2 (95% CI, 1.8–4.6; *p* < 0.001).	The use of alcohol-based hand foam can simultaneously disinfect the hands and the stethoscope diaphragm
Wood M et al. (2007)	U.S.A.	2003	A medical/surgical/trauma intensive care unit (ICU) and a regional trauma emergency department (ED)	Cross-sectional study	To test the utility of the stethoscope covers impregnated with silver ions in preventing surface contamination	74 (37 with cover, 37 no cover)	*Stethoscopes*	-	The mean number of CFU: uncovered: 71.4 cover < 1 week: 246.5 cover > week: 335.6	The use of the cover was associated with significantly higher colony counts
Hill et al. (2005) *Letter to editor*	U.K.	n.r.	Elderly care department	Prospective, cross-sectional study pre/post design	To evaluate the effectiveness of both the sensitization campaign and of the increased availability of alcohol wipes	n.r.	*Stethoscopes*	-	The mean total colony count: baseline: 70, one month: 59, three months: 41 (↓ 41%) MRSA colonies fell from 0.42 per stethoscopes to 0.08 per stethoscopes at one month (↓83%). No MRSA colonies at three months (↓100%)	-The awareness campaign was effective -the alcohol wipes decrease the bacterial load - No MRSA colonies at three months
Parmar et al. (2004)	India	n.r.	Tertiary care hospital	Prospective randomized, double blind study	To determine the effectiveness of disinfection with 66% ethyl alcohol	100	*Stethoscopes*	94.8 (*Group B*)	Before cleaning: 90% stethoscopes contaminated - Immediately after cleaning: 28% stethoscopes contaminated after five days without cleaning: 95% stethoscopes contaminated -Five days after cleaning once a day: 25% stethoscopes contaminated	66% ethyl alcohol was an effective disinfectant
Leprat et al. (1998) *Letter to editor*	France	n.r.	Service d’Higiene Hospitaliere	Cross-sectional study pre/post design	To assess: - The efficacy of wipes impregnated with benzalkonium to clean stethoscopes -The rate of recontamination after use	105	*Stethoscopes*	100	Before disinfection: 97 stethoscopes contaminatedAfter disinfection: No stethoscopes contaminated The rate recontamination: after five uses ->100% recontaminated stethoscopes	-The reduction of the bacterial load was remarkable -The rate of recontamination increases with increasing the use
Marinella M et al. (1997)	U.S.A.	n.r.	Intensive care unit	Cross-sectional study pre/post design	To compare the effectiveness of various cleaning agents	40 (10 for each method tested)	*Stethoscopes*	≥80.6 (IPA, NaOCl, BAK)	The mean (±SE) of CFU and: Before disinfection diaphragm 158 ± 33 rim 289 ± 54 After disinfection:diaphragm: 0.2( ± 0.2) (*p* = 0.2) IPA 0.1(±0.1) (*p* = 0.2) NaOCl 0.6(±0.4) (*p* = 0.02) BAK 47(±28) (*p* = 0.11) Soap and water Rim: 2.2(±1.5) (*p* = 0.01) IPA 50(±29) (*p* = 0.04) NaOCl 56 ± 49 (*p* = 0.04) BAK 95 ± 48 (*p* = 0.11) Soap and water	-The most cleaning agent was IPA. -In addition, sodium hypochlorite and benzalkonium chloride significantly decreased colony counts. Soap and water did not do it significantly

PedsED: Pediatric Emergency Division; AICU: Adult medical intensive care unit; CFU: Colony Forming Unit; IQR: Interquartile Range; LED: Light-Emitting Diode; UVC: Ultraviolet C; HAI: Healthcare-associated infections; EBHS: Ethanol- Based Hand Sanitizer; MRSA: Methicillin-Resistant Staphylococcus Aureus; MR-CNS: CNS Resistant to Methicillin; IPA: Isopropyl alcohol; NaOCl: Sodium hypochlorite; BAK: Benzalkonium chloride; TBC: Total Bacterial Count; SE: Standard Error.
